# Percutaneous Ultrasound-Guided Laser Ablation with Contrast-Enhanced Ultrasonography for Hyperfunctioning Parathyroid Adenoma: A Preliminary Case Series

**DOI:** 10.1155/2015/673604

**Published:** 2015-12-16

**Authors:** Tianan Jiang, Fen Chen, Xiang Zhou, Ying Hu, Qiyu Zhao

**Affiliations:** ^1^Department of Ultrasonography, The First Affiliated Hospital, College of Medicine, Zhejiang University, Qingchun Road No. 79, Hangzhou, Zhejiang 310003, China; ^2^Hepatobiliary & Pancreatic Intervention Center, The First Affiliated Hospital, College of Medicine, Zhejiang University, Qingchun Road No. 79, Hangzhou, Zhejiang 310003, China; ^3^Department of Ultrasound, West China Hospital, Sichuan University, Guoxue Xiang No. 37, Wuhou, Chengdu, Sichuan 610041, China

## Abstract

The study was to evaluate the safety and effectiveness of ultrasound-guided percutaneous laser ablation (pLA) as a nonsurgical treatment for primary parathyroid adenoma. Surgery was contraindicated in, or refused by, the included patients. No lesion enhancement on contrast-enhanced ultrasound immediately after pLA was considered “complete ablation.” Nodule size, serum calcium, and parathyroid hormone level were compared before and after pLA. Complete ablation was achieved in all 21 patients with 1 (*n* = 20) or 2 (*n* = 1) sessions. Nodule volume decreased from 0.93 ± 0.58 mL at baseline to 0.53 ± 0.38 and 0.48 ± 0.34 mL at 6 and 12 months after pLA (*P* < 0.05). At 1 day, 6 months, and 12 months after pLA, serum PTH decreased from 15.23 ± 3.00 pmol/L at baseline to 7.41 ± 2.79, 6.95 ± 1.78, and 6.90 ± 1.46 pmol/L, serum calcium decreased from 3.77 ± 0.77 mmol/L at baseline to 2.50 ± 0.72, 2.41 ± 0.37, and 2.28 ± 0.26 mmol/L, respectively (*P* < 0.05). At 12 months, treatment success (normalization of PTH and serum calcium) was achieved in 81%. No serious complications were observed. Ultrasound-guided pLA with contrast-enhanced ultrasound is a viable alternative to surgery for primary parathyroid adenoma.

## 1. Introduction

Primary hyperparathyroidism (HPT) is the most common cause of chronic hypercalcemia, with an incidence of 0.03% in the general population; furthermore, its incidence increases with age [1]. Long-standing HPT is associated with complications such as renal stones, osteoporosis, and bone pain. In addition, it has been reported that primary HPT is associated with hypertension, disturbances in the renin-angiotensin-aldosterone system, vascular wall changes, and left ventricular hypertrophy [2]. The most common cause of primary HPT is an adenoma, and in 80–90% of patients this can be identified by ultrasound (US) scanning as a single adenoma [3]. 

Surgery is the recommended treatment for patients with symptomatic primary HPT due to the high long-term cure rate [4]. Many patients with primary HPT can be treated with minimally invasive parathyroidectomy under sedation and local anesthesia, thereby eliminating the need for general anesthesia and its associated risks. Nonetheless, surgery is not without its risks and complications, and these may be increased in older patients, particularly if comorbidities are present. As a result, some patients of advanced age may decline the surgical approach due to the perceived higher surgical risks. Some younger patients are also unwilling to undergo resection for cosmetic reasons, since this would leave a surgical scar on the neck. The availability of an alternative management strategy would therefore benefit the minority of patients who decline surgery or for whom surgery is considered inappropriate.

Several techniques have been investigated as alternatives to surgery for the treatment of primary HPT, including percutaneous ethanol injection (PEI), radiofrequency ablation (RFA), high-intensity focused US (HIFU), and thermal ablation techniques. Laser ablation (LA) was proposed over a decade ago as a possible therapy for patients with thyroid and parathyroid nodules [5, 6]. In one study, it was reported that the necrosis zone could be controlled precisely through regulation of the output energy of the laser, resulting in no or only minimal damage to the surrounding tissue [6]. However, another investigation determined that LA could not be used as a definitive therapy for primary HPT, because complete thermal destruction of all cells within the nodule was difficult to achieve for parathyroid adenomas [7].

In order for LA to become a more widely accepted alternative to surgery for primary HPT, the technique must be improved to avoid incomplete ablation and hence relapse. With this in mind, the aim of the present study was to assess whether one-stage complete ablation could be maximally achieved in patients with primary HPT through the use of contrast-enhanced ultrasonography (CEUS) to observe the ablation area immediately after LA.

## 2. Patients and Methods

### 2.1. Patients

This was a retrospective study of consecutive patients newly diagnosed with primary HPT secondary to adenoma, treated between January 1, 2010, and November 1, 2012, at The First Affiliated Hospital of Zhejiang University, China. The study was performed in accordance with the Declaration of Helsinki and was approved by the institutional review board of The First Affiliated Hospital of Zhejiang University. All patients provided informed written consent for treatment with LA therapy.

The inclusion criteria were as follows: (1) age ≥ 18 years; (2) a serum calcium concentration ≥ 2.60 mmol/L; (3) a parathyroid hormone (PTH) level higher than the normal range (0.9–8.2 pmol/L); (4) a single parathyroid adenoma (volume > 0.1 mL) detected clearly by US; (5) US-guided fine-needle (21-gauge) aspiration provided pathological confirmation that the lesion was of parathyroid origin [8]; and (6) surgical treatment that was contraindicated (because of a high risk of general anesthesia or symptoms of acute severe hypercalcemia) or refused (to avoid a postoperative scar on the neck).

The exclusion criteria were (1) multiple endocrine neoplasia; (2) concomitant vitamin D deficiency (serum 25-hydroxy-vitamin D < 20 ng/mL); (3) nodule volume > 2.5 mL; (4) multiple nodules in both sides of the neck; and (5) prior neck surgery/parathyroidectomy or familial hypocalciuric hypercalcemia.

### 2.2. LA Procedures

In order to maximize the possibility of complete necrosis and reduce the incidence of complications, ultrasonography (Esaote, MyLab 90, LA 523, Italy) and CEUS with rapid bolus injection of contrast agent (2.4 mL of sulfur hexafluoride; SonoVue; Bracco SpA, Milan, Italy) were performed before LA to determine the size and blood supply of the parathyroid gland and evaluate the surrounding tissues. Parathyroid nodule volume was calculated according to the equation *V* = *πabc*/6, where *a* represents the largest diameter and *b* and *c* represent the other two perpendicular diameters [7]. The measurements were made three times by the same investigator (who had over 20 years' experience in sonographically guided interventional procedures for thyroid tumors), and the mean value was calculated.

LA was performed by the same investigator, who had over 15 years' experience of US-guided ablation treatment. The procedure was carried out under monitored anesthesia care with intravenous infusion of dexmedetomidine (loading dose 1 *μ*g/kg over 10 min, followed by a maintenance dose of 0.2–0.7 *μ*g/kg/h). The patient was conscious throughout the procedure to allow the assessment of vocal cord function: should any patient experience hoarseness of voice during the procedure, the LA would be stopped immediately and laryngoscopy performed (during the treatment session) to assess vocal cord mobility. In addition, the patient was asked to report any discomfort during the LA procedure, using a validated visual-analog scale (from 1 = no pain to 10 = intolerable pain).

Local anesthetic (lidocaine 2%) was administered under US guidance, via a 22-gauge Chiba needle (Top, Tokyo, Japan), to the skin, subcutaneous tissues, muscles, and tissues adjacent to the parathyroid gland, particularly those situated posteriorly. Then, a 21-gauge Chiba needle (Top, Tokyo, Japan) was placed carefully into the target parathyroid lesion, using a freehand-guided technique.

The needle core was removed, and a plane-cut quartz optic fiber (diameter, 300 *μ*m) was advanced through the needle sheath and positioned in the parathyroid nodule. The sheath was withdrawn to leave the bare optic fiber tip in direct contact with the parathyroid tissue. The LA procedure was performed with an output power of 3.0 W (Echolaser X4, ESAOTE, Italy), delivered for 3–10 min depending on lesion volume (3 min for lesions < 1 mL in volume, and increasing progressively with lesion volume up to a maximum of 10 min). The central portion of the nodular area was monitored continuously with ultrasonography to prevent overablation. The LA session was ended when the entire parathyroid gland had developed hyperechogenicity.

**Figure 1:**
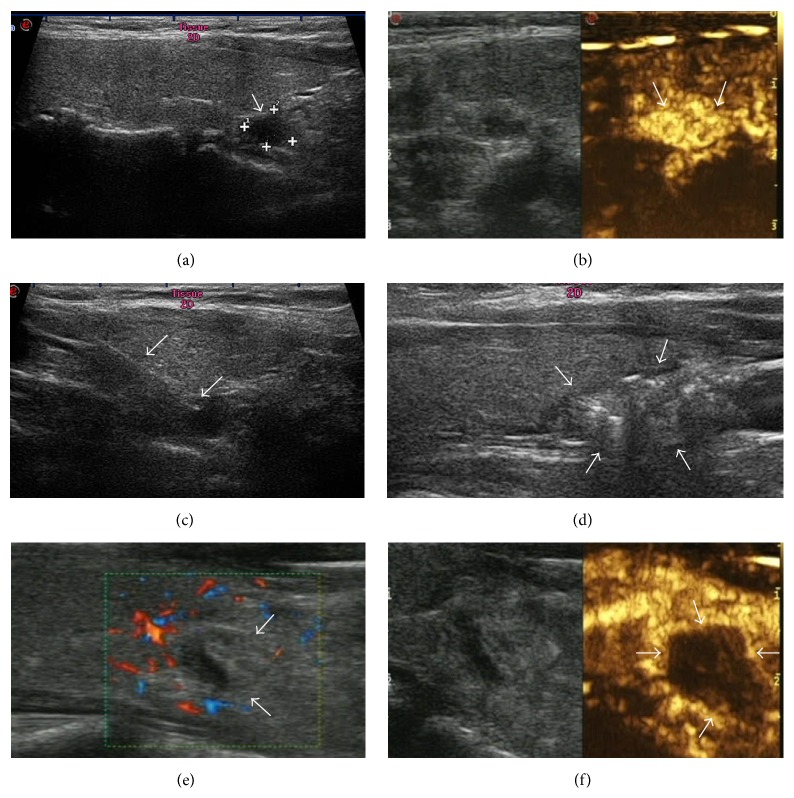
Representative ultrasonography images from one patient, a 45-year-old woman with parathyroid adenoma and hyperparathyroidism, illustrating the LA procedure. (a) Longitudinal section of the right neck revealed a 0.21 mL enlarged parathyroid gland (arrow and cursors) situated posterior to the inferior portion of the right lobe of the thyroid gland. (b) Preablation CEUS demonstrated hyperenhancement of the parathyroid lesion. (c) Ultrasonography image showing a 21-gauge needle inserted into the parathyroid; the arrows point to the needle tip. (d) During the LA procedure, the tissue around the fiber tip became hyperechoic (arrows) under US monitoring, and the hyperechoic area gradually enlarged until the nodule became filled with hyperechogenicity. This LA process was repeated throughout the parathyroid gland until most of gland had been ablated. (e) The color Doppler image obtained 1 hour after ablation showed no flow signal in the ablated area (arrows). (f) After the procedure had been completed, CEUS showed no enhancement of the ablated area by the contrast agent (arrow).

CEUS was performed 10 min after LA to assess the vascularity of the nodule. If partial intranodule enhancement was observed by CEUS, an additional LA session was conducted immediately with the aim of obtaining a lack of enhancement throughout the entire nodule. In some patients, color Doppler ultrasonography was performed 1 hour after completion of the procedure to determine whether a blood flow signal could be detected. Representative ultrasonography images from one patient, illustrating the LA procedure, are shown in Figure 1.

### 2.3. Follow-Up and Outcome Measures

A validated visual-analog scale (from 1 = no pain to 10 = intolerable pain) was used to assess the pain levels experienced by each patient. Patients were monitored closely during the 24 h after percutaneous LA for possible complications such as fever, skin burns, and pain.

Serum biochemical profiles, including parathyroid hormone levels (reference range, 0.9–8.2 pmol/L) and serum calcium levels (reference range, 2.12–2.60 mmol/L), were obtained before the first LA session and 1 day, 6 months, and 12 months after the LA session. Follow-up US was performed 6 months and 12 months after LA therapy to evaluate changes in parathyroid gland size.

The effectiveness of the treatment at 6 months and 12 months after LA was evaluated from changes in parathyroid gland size, serum PTH level, and serum calcium concentration. Normal levels of serum calcium and PTH at 12 months after LA were considered to be “treatment success.” Reduced adenoma volume and/or decreased PTH secretion and serum calcium concentration ≤ 0.25 mmol/L above the upper reference range (i.e., ≤2.85 mmol/L) were defined as “good control.” Treatment was considered “unsuccessful” when no reduction in parathyroid volume or PTH level was achieved, and serum calcium concentration persisted > 0.25 mmol/L above the upper reference range [9].

### 2.4. Statistical Analysis

Continuous variables are presented as the mean ± standard deviation (SD). Statistical comparisons were made using one-way analysis of variance (ANOVA) with a Student-Newman-Keuls (SNK) post hoc test. *P* < 0.05 was considered statistically significant. SPSS 10.1 software (Chicago, IL, USA) was used for the statistical analyses.

## 3. Results

### 3.1. Patient Characteristics

A total of 21 patients (aged 33 to 63 years) with primary HPT secondary to parathyroid adenoma were enrolled in this study. The demographic and clinical characteristics of these patients are presented in Table 1. The majority of patients were aged ≥ 50 years, and the female : male ratio was 5 : 2. Seventeen patients were not recommended for surgical treatment due to a high risk of general anesthesia or symptoms of acute severe hypercalcemia, while 4 patients refused surgery to avoid a postoperative scar on the neck.

### 3.2. Laser Ablation Treatment

Complete ablation (i.e., a lack of enhancement shown by CEUS, which was performed 10 min after LA to assess nodule vascularity) was achieved successfully in all 21 patients. Twenty patients (95.2%), each with a single nodule < 2.0 mL in volume, required only one LA session to achieve complete ablation. In these patients CEUS demonstrated an absence of vascularization in the treated area 10 min after the ablation. The remaining patient, with a nodule 2.49 mL in volume, required 2 LA sessions (13.9 min duration, 2502 J total output) that were carried out on the same day, due to CEUS showing partial enhancement of the lesion after the first LA session. The mean treatment duration was 9.66 ± 2.19 min (range, 3.0–13.9 min) and depended on the nodule size (range, 0.10–2.49 mL). The mean total energy delivery, calculated as laser power (3 W) multiplied by duration of exposure, was 1739 ± 394 J (range, 540–2502 J).

### 3.3. Follow-Up and Outcome Measures

The follow-up period ranged from 12 to 16 months (mean, 13.19 ± 0.98 months). At the 1-year follow-up, “treatment success” was achieved in 17/21 patients (81%), while the other 4 patients (19%) were considered to have their tumor under “good control” (as defined above in Patients and Methods).

All patients exhibited a reduction in parathyroid gland volume after LA (Table 2). The mean parathyroid gland volume decreased from 0.93 ± 0.58 mL at baseline to 0.53 ± 0.38 mL at 6 months after LA (*P* < 0.05) and 0.48 ± 0.34 mL at 12 months after LA (*P* < 0.05). This corresponded to a mean reduction in parathyroid gland volume of 43.3 ± 20.8% at 6 months after LA and 45.1 ± 34.1% at 12 months after LA.

The mean serum PTH level decreased after LA treatment (Table 2), from a value of 15.23 ± 3.00 pmol/L at baseline to values of 7.41 ± 2.79 pmol/L at one day after LA, 6.95 ± 1.78 pmol/L at 6 months after LA, and 6.90 ± 1.46 pmol/L at 12 months after LA (all *P* < 0.05 versus baseline).

There was also a reduction in the mean serum calcium concentration after LA therapy (Table 2), from 3.77 ± 0.77 mmol/L at baseline to 2.50 ± 0.72 mmol/L at one day after LA, 2.41 ± 0.37 mmol/L at 6 months after LA, and 2.28 ± 0.26 mmol/L at 12 months after LA (all *P* < 0.05 versus baseline).

### 3.4. Complications

All patients reported a “burning” sensation during the ablation procedure. However, this discomfort was tolerated without the need for additional anesthesia and was rated by all patients as <3 on the visual-analog scale. In the 6 hours after LA therapy, 2/21 patients (9.5%) described transient cervical pain that was rated as grade 2/10; no administration of analgesia was required. Although LA of the parathyroid is associated with a risk of recurrent laryngeal nerve injury, only 1 patient in our study exhibited recurrent laryngeal nerve palsy, and this occurred during the first 3 days following LA; full recovery was achieved after one week without the need for any clinical treatment. No permanent paralysis was observed in any of the patients. Furthermore, there were no incidences of neck hematoma, infection, burn injuries, or hypocalcemia. There were no significant long-term complications from the LA therapy.

## 4. Discussion

The main findings of the present study were that US-guided percutaneous LA therapy in patients with primary HPT due to parathyroid adenoma resulted in significant reductions in nodule size, serum calcium concentration, and serum PTH level that were maintained for 12 months. Furthermore, therapy was optimized by using CEUS to assess the ablated area immediately after LA. In addition, the procedure was associated with only transient adverse effects, with no serious complications or deaths during the 12-month follow-up. Therefore, US-guided percutaneous LA may represent a useful treatment option in patients with hyperfunctioning parathyroid adenoma when surgery is either refused or contraindicated.

Only a small number of case studies have assessed LA for the management of parathyroid adenoma. The first of these described the successful use of LA in a single patient with primary HPT [5]. However, a subsequent investigation reported that only 1 of 3 patients showed long-term remission of HPT after LA [10]. A more recent study of 6 patients found that although LA was safe, it achieved only short-lived reductions in serum PTH and calcium levels [7]. Important limitations of all these studies are their small sample sizes and failure to image the ablation area after LA to determine whether complete ablation had been achieved. In thyroid tissue, US images have been reported not to correlate with histological assessments of coagulation area, suggesting that standard US imaging can only provide a rough guide to the actual extent of the coagulation zone [11]. Although magnetic resonance imaging can provide accurate monitoring of laser-induced tissue damage, there are as yet no suitable devices for this particular application. The present investigation not only enrolled a much larger number of patients (21) than previous case studies of LA for HPT, but also utilized CEUS to confirm complete ablation of the target area. Thus, despite some negative findings in previous reports, the present study provides evidence that LA is a useful nonsurgical option for patients with parathyroid adenoma, particularly when complete ablation is established using CEUS.

The use of CEUS to assess the ablation area immediately after LA helped to optimize the ablation result, and we consider this to be an important and novel aspect of our study. CEUS can reveal microvascularization of an organ and has been widely used to evaluate the completeness of liver tumor ablation and thyroid ablation. We believe that CEUS carried out within minutes of LA can give a good indication as to whether the ablation is complete or partial, making it possible to perform an additional ablation (if required) and complete the treatment in the same operative session. Nonetheless, an absence of residual viable tissue detected by CEUS does not guarantee complete destruction of the adenoma or death of all cells of the parathyroid tissue; longer-term follow-up (e.g., for 36 months or more) showing sustained normalization of PTH and calcium levels would be required to confirm complete ablation. It may be that the detection by CEUS of an ablation area larger than the initial volume of the adenoma would make it more likely that complete ablation had been achieved.

Although surgical parathyroidectomy remains the definitive therapy for symptomatic primary HPT [12, 13], nonsurgical alternatives are available for patients in whom surgery is refused or contraindicated. Medical therapies include calcimimetics (such as cinacalcet) [14], but these are expensive and can cause gastrointestinal disturbances [15]. Nonsurgical ablation techniques include arteriographic ablation, PEI, RFA, HIFU, and LA. Arteriographic ablation requires sophisticated operator skill, so its use is restricted to a few centers with appropriate expertise [16]. Although PEI can be easily performed and causes tumor necrosis and shrinkage [17], its use in parathyroid adenoma is limited by several disadvantages, including nonuniform diffusion within the tissue, high local recurrence rates (necessitating repeat treatments), and fibrosis due to ethanol leakage outside the nodule capsule that impedes subsequent surgery [18–20]. Studies of RFA for thyroid and parathyroid tumors have been limited to case reports and small-series cases [21, 22]. Moreover, the RFA needle (17 gauges) is much larger than that required for LA, increasing the risk of bleeding and injury to surrounding tissues, including the recurrent laryngeal nerve. Few reports have described the use of HIFU for the management of parathyroid disease due to its complexity and time-consuming nature [23]. The latest study of HIFU ablation for HPT (13 patients) revealed that the method was safe and achieved good disease control in most patients; however, complete remission after one year was achieved in only 23% of patients [9].

Important advantages of LA over other techniques are precise control of the ablation parameters and the ability to achieve a small and precise ablative area using small needles, thereby minimizing damage to surrounding tissues. For this reason and in view of the limitations of HIFU ablation, LA seems to be the ideal ablative method for treating benign or even malignant tumors in a complex anatomical region such as the neck. In our study, treatment success was achieved in 81% of patients, higher than that obtained with HIFU ablation [9], and the entire procedure required only 9.1 ± 3.0 min (comparable to that for RFA). Furthermore, complete ablation of the nodule was confirmed by postprocedural CEUS, and the volume reduction was maintained during the 12-month follow-up. An additional advantage was that the procedure was well tolerated, with only a low grade of transient discomfort. Temporary nerve palsy occurred in only one of the 21 patients (who had the largest nodule volume and required two sessions of LA). Based on our results, we suggest that small lesions (<1.75 mL) can be treated successfully with a single ablation procedure, whereas repeated sessions may be necessary for larger nodules.

The present study is not without limitations. First, the sample size was small, necessitating further studies of larger cohorts. Second, the follow-up period was only 12 months; thus, it was not possible to establish the longer-term efficacy of the procedure or possible long-term complications. Third, it was not possible to determine the optimal parameters for LA (i.e., laser power, duration of exposure, and total energy delivered). Fourth, comparisons of one versus several ablation procedures were not undertaken. Nonetheless, our study provides an important foundation for the design of future investigations to assess the clinical effectiveness of this management technique. Additionally, large-sample, prospective, randomized controlled trials, with a longer follow-up, are merited to compare the safety and efficacy of LA therapy with surgical management of parathyroid adenoma.

In conclusion, US-guided percutaneous LA was successfully performed in 21 patients with primary parathyroid adenomas, with CEUS used to visualize the ablation zone immediately after LA in order to help optimize the ablation procedure. The results of this study suggest that LA may be a safe, well-tolerated, and effective therapy for the treatment of primary parathyroid adenoma in selected patients who are not amenable to surgery or who refuse surgery. Furthermore, imaging of the ablated area with CEUS played an important role in the achievement of complete ablation.

## Figures and Tables

**Table 1:** Demographic and clinical characteristics of the 21 patients enrolled in the study.

Demographics	
Age < 50 years (*n*)	4
Age ≥ 50 years (*n*)	17
Sex (male/female)	6/15
Clinical manifestations	
Osteoporosis (*n*)	9
Asymptomatic (*n*)	5
Nephrolithiasis (*n*)	7
Tumor size	
Mean volume (mL)	0.94 ± 0.13
Volume < 1.0 mL (*n*)	13 (minimum volume, 0.10 mL)
Volume ≥ 1.0 mL (*n*)	8 (maximum volume, 2.49 mL)
Tumor location	
Left inferior (*n*)	13
Left superior (*n*)	0
Right inferior (*n*)	8
Right superior (*n*)	0
Laser ablation sessions	
1 session (*n*)	20
2 sessions (*n*)	1

Mean tumor volume presented as mean ± standard deviation.

**Table 2:** Tumor volumes, serum parathyroid hormone levels, and serum calcium concentrations before and after laser ablation therapy.

Outcome measure	Before LA	1 day after LA	6 months after LA	12 months after LA
Tumor volume (mL)	0.93 ± 0.58	—	0.53 ± 0.38^*∗*^	0.48 ± 0.34^*∗*^
Serum PTH (pmol/L)	15.23 ± 3.00	7.41 ± 2.79^*∗*^	6.95 ± 1.78^*∗*^	6.90 ± 1.46^*∗*^
Serum calcium (mmol/L)	3.77 ± 0.77	2.50 ± 0.72^*∗*^	2.41 ± 0.37^*∗*^	2.28 ± 0.26^*∗*^

Data are presented as the mean ± standard deviation. LA: laser ablation; PTH: parathyroid hormone; —: not measured. Normal ranges: serum PTH, 0.9–8.2 pmol/L; serum calcium, 2.12–2.60 mmol/L. ^*∗*^ *P* < 0.05 versus before LA.
